# Effect of Graphene Oxide on Liquid Water-Based Waterproofing Bituminous Membranes

**DOI:** 10.3390/polym14112221

**Published:** 2022-05-30

**Authors:** Giuseppe Di Luca, Marcello Filomia, Alessio Fuoco, Giovanni Chiappetta, Alberto Figoli

**Affiliations:** 1 Institute on Membrane Technology, National Research Council of Italy (CNR-ITM), via P. Bucci 17/C, 87036 Rende, Italy; g.diluca@itm.cnr.it (G.D.L.); g.chiappetta@itm.cnr.it (G.C.); 2 Research and Developed Department, HA ITALIA S.p.A, Viale della Scienza 78, 36100 Vicenza, Italy; marcello.filomia@ha-italia.com

**Keywords:** graphene oxide (GO), mechanical properties, resistance to aging, liquid bituminous waterproofing, water vapor transport

## Abstract

In this work, innovative graphene oxide–doped waterproofing bituminous membranes, also called roof bituminous membranes, were prepared and characterized in terms of physicochemical and vapor transport properties. The results showed that the introduction of a small amount of GO increased the mechanical resistance of the doped membranes compared to the native one. Moreover, the addition of the GO leads to a remarkable chemical stability of the membranes when exposed to UV radiation and high temperatures. Furthermore, a decrease in water vapor permeation was observed when GO was present in the membrane matrix compared to native bituminous membranes, demonstrating that an addition of GO can boost the waterproofing properties of these bituminous membranes.

## 1. Introduction

Rising damp, humidity, and water, in general, cause irreversible structural and aesthetic damages to buildings [1]. In fact, water infiltrations transport chloride ions into the reinforced concrete, triggering the corrosion of the steel reinforcement, and the produced rust causes the concrete spalling [1,2]. In this context, bituminous membranes are widely used in many countries as a waterproofing layer to create a barrier to water and humidity for the protection of roofs, terraces, retaining walls, and any other kind of civil and industrial surfaces [3].

Bitumen is a complex and variegated mixture of compounds that includes, but it is limited to, hydrocarbons (e.g., polycyclic aromatic and saturated hydrocarbons) and various trace metals. When used as a protective and insulating layer, the bitumen membrane’s lifetime is reduced by aging phenomena due to heat, UV radiation, and atmospheric agents, both during production processes and after installation [4]. Its aging is mainly caused by oxidation [5,6,7], which modifies the chemical structure of the bitumen by the increase of carbonyl and sulfoxide groups as a function of the degree of aging [8,9]. This results in an irreversible increase in the molecular weight of the ligand related to the formation of macromolecules and a radical modification of its colloidal structure, phenomena that compromise its performance [10,11,12]. Thermo-oxidative and photo-oxidative aging phenomena depend on the different sources of excitation to generate free radicals in the initial phase: heat and ultraviolet radiation, respectively [13,14]. Strategies for preventing the degree of aging of bitumen consist of reducing the excitation energy and the contact with oxidizing agents, such as oxygen [15] 

A commonly used strategy in bitumen waterproofing membrane manufacturing technology for performance enhancement includes the addition of polymers such as EPDM (Ethylene-Propylene-Diene Monomer) [16], CPE (Chlorinated Polyethylene) [17], PE (polyethylene) [18], PP (polypropylene) [19], EVA (ethylene–vinyl acetate) [20], EBA (ethylene–butyl acrylate) [21], SBS (styrene–butadiene–styrene) [22], SIS (styrene–isoprene–styrene) [23], SEBS (styrene–ethylene/butylene–styrene) [24], KEE (Ketone Ethylene Ester), plasticized PVC, neoprene, and others [25,26]. This addition leads to the enhancement of bitumen properties in terms of stiffness at high temperatures, low-temperature cracking resistance, resistance to humidity, and resistance to fatigue. However, the main limitations in using those additives are the high costs and low resistance to aging [25]. The latter is particularly important since the polymer-doped membranes are unable to resist the degradation effect produced by environmental conditions such as UV radiation, heat, and atmospheric conditions [27], especially because it often occurs before the end of the expected lifetime [28].

Another class of possible additives to be used for improving the performance of bitumen are nanomaterials, such a nano-clays, nano-silica, and nano-carbon fiber [29], since they can improve its rheological properties [30,31]. Moreover, nanomaterials with a layered structure, such as bilayer lamellar hydroxides (LDHs) [32], montmorillonite [33], and carbon black [34], can be used as anti-aging agents for bitumen, due to their oxygen barrier function. However, the dispersion of these materials in the bituminous matrix is very difficult, since they easily form aggregates that lead to a non-uniform distribution in the matrix.

In this class of materials, graphene oxide (GO) presents a high dispersibility in the bituminous matrix, since it is structurally and chemically similar to asphaltenes, which are among the main components of bitumen [35]. The graphene oxide molecular structure is analogous to that of graphene, but with oxygen-containing functionalities such as hydroxyl, carbonyl, and epoxide groups on the basal layers and on the edges of the lamellar [36,37], that confer the good compatibility with both hydrophilic and hydrophobic materials [6,38]. Generally, the high cost of the GO [39] represents a limitation in low-cost materials such as bitumen and bituminous membranes, which are the products widely used in the construction field.

It has been reported the inclusion of GO in bituminous matrices provided an improvement in terms of the mechanical strength [40], as well as in terms of the reduction of gas and liquid transport [41]. Moreover, small quantities of GO improve the resistance to thermal oxidation and UV light and give greater tenacity to fatigue cracking and permanent deformation [42].

This work is the first time that, to the best of our knowledge, small quantities of multilayer GO were used to produce high-performing liquid bituminous membranes with a waterproofing function. A low GO concentration was selected according to the literature [43,44] in order to have a low impact on production cost and, thus, to be competitive in the target market. Therefore, this study aimed to analyze the effect of the low graphene oxide concentration on the mechanical resistance of the produced membrane, how the GO doping impacts the barrier properties, and its properties as an anti-aging agent.

## 2. Experimental Section

### 2.1. Materials

A road bitumen with 70/100 dmm penetration, from the Alma Petroli S.p.A. (Ravenna, Italy) refinery, was used as a bituminous binder, and its properties are reported in Table 1. This product is used for the production in the laboratory of an anionic (basic) bituminous emulsion, which allows researchers to obtain the water-based liquid waterproofing.

Graphene oxide powder, 15–20 sheets, 4–10% edge oxide, was purchased from Sigma Aldrich, and it was dispersed and exfoliated in aqueous soapy solution.

### 2.2. Methods

#### 2.2.1. Preparation of GO-Modified Emulsion

Three samples of basic bituminous emulsion with 55% of road bitumen with 70/100 dmm penetration were prepared: a reference sample or native membrane and two other membranes loaded with two different percentages of GO, 0.04 wt.% (MGLQ) and 0.12 wt.% (MGHQ), based on the final weight of the self-standing membrane.

**Figure 1:**
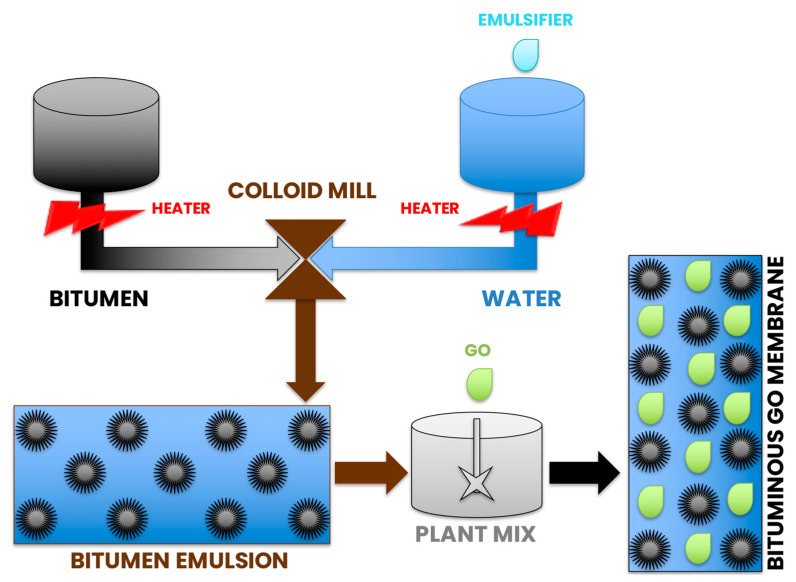
Bituminous emulsion preparation scheme.

In this work, GO was dispersed in water by using the sonication method [45], and in order to exfoliate and disperse the GO, a procedure comprising of 20 sonication/shaking cycles of 30 minutes was used. The aqueous solution was used to produce the bituminous emulsion, using a colloidal mill at high speed (3000 rpm). Figure 1 reports a schematic representation of the preparation route. 

#### 2.2.2. Preparation Bituminous Membranes

The bituminous membranes were prepared by casting the bituminous emulsion in a 1 mm–thick rectangular mold with the dimensions 25 cm × 10 cm, using a knife. The membranes were then left to dry at room temperature (20 ± 2 °C) for 7 days. 

#### 2.2.3. Viscosity Measurement

The viscosity of the doped GO emulsions was measured by using a rotational viscosimeter DV1 Digital Viscometer (Brookfield Ametek, Middleborough, MA, USA) equipped with a thermostatic bath. The measurements were acquired at 20 °C and repeated three times, employing a spindle n°6.

#### 2.2.4. ATR–FTIR Characterization

The IR spectra of the membrane surfaces were characterized with the instrument UATR crystal Diamond/ZnSe–Spectrum One System, (Perkin Elmer Instruments). For every sample, acquisitions at three different surface spots were collected in the wavenumber range from 4000 to 650 cm^−1^ and at a resolution of 4 cm^−1^.

#### 2.2.5. Accelerated Weathering Tester (QUV)

The damages caused by exposure to atmospheric agents in medium–long periods were simulated by an accelerated aging process, using the QUV technique [46], i.e., by irradiation of the samples with high-temperature UV rays for a period of 1000 h (about 42 days). 

#### 2.2.6. Surface Property Characterization

The hydrophilicity of the membranes was quantified by water contact angle (WCA), using the Young–Laplace method of the sessile drop (CAM 200 Optical contact angle meter), employing a procedure already reported in the literature [47]. At least five acquisitions were performed for each sample, and the angles were measured at the immediate droplet release to avoid errors due to evaporation phenomena. The surface morphology of the membrane was studied with a scanning electron microscope (SEM) (EVO MA10, Zeiss, Milan, Italy), and the images were acquired in high vacuum mode on previously gold-coated samples (Quorum Q150 RS, Quorum Technologies, Lewes, UK).

#### 2.2.7. Mechanical Properties

The mechanical properties of the membranes were measured by using a tensile analyzer (Roell/Zwick universal testing machine, single column Z2.5). At least five measurements were performed on each sample at room temperature, the starting distance of the clamps was set at 30 mm, and the samples deformation rate was set at 2 mm min^−1^.

#### 2.2.8. Permeability

The water permeability vapor was determined by the cup test method, with the gravimetric method, according to ASTM E at 20, 40, and 60 °C. Each test was performed three times for a period of 48 h in a desiccator to maintain dry condition. The mass flux, *F* (g m^−2^ h^−1^), of the water vapor through the self-standing bituminous membranes was determined by the following equation [48]:

(1) $$F=\frac{1}{A}\frac{dm_{Loss}}{dt}$$

where *A* is the active exposed area of the membrane (m^2^), and *m_Loss_* is the water mass loss from the container (g) in a defined unit of time (h). In order to compare the material performances of bituminous membranes with different thicknesses, *d* (m), the F_d_ (g m m^−2^ h^−1^) theoretical value is herein introduced by multiplying F by the thickness of the self-standing bituminous membranes.

## 3. Results and Discussion

### 3.1. Properties of Bituminous Emulsion

The properties of the native bituminous solution fulfill all the requirements for this class of materials (Table 2), according to the European standards, and due to the low GO content, the other two solutions are also believed to be in the same range of values. This statement is supported by the viscosity measurements (Table 3), which are not influenced by the addition of low quantities of GO. The emulsion was used to produce stable and self-standing bituminous membranes of approximately 1 mm in thickness that were possible to handle without particular precautions.

### 3.2. ATR–FTIR

**Figure 2:**
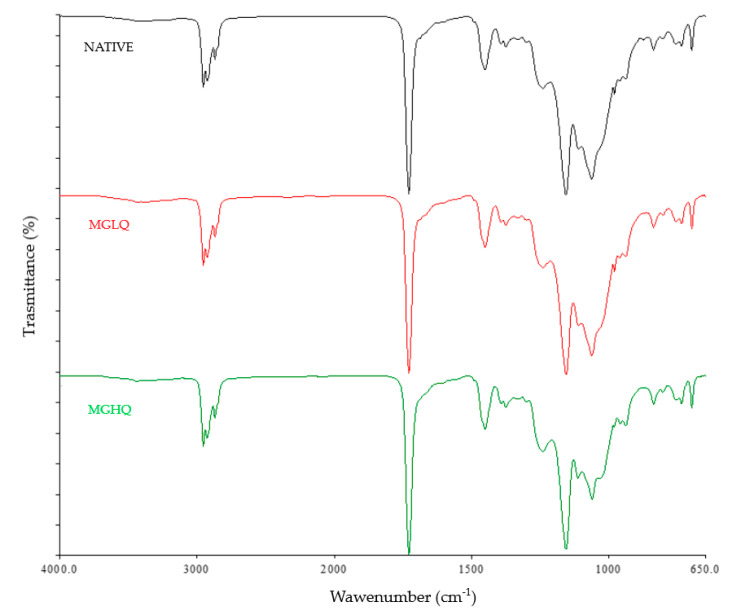
ATR–FTIR spectrum acquired for the native (black), MGLQ (red), and MGHQ (green) membranes.

The IR spectra acquired in the ATR–FTIR mode show the typical fingerprint of bituminous materials (Figure 2). The absorption peaks at 2958, 2938, and 2873 cm^−1^ are characteristic of the C–H bond stretching vibration peak of alkanes, methylene, and naphthenes, respectively. The strong absorption peak at 1730 cm^−1^ is a typical signal of aromatic ketone. The absorption peaks at 1453 and 1377 cm^−1^ are due to the bending vibration of methylene and methyl group, respectively, and the peak at 1064 cm^−1^ is due to the sulfoxide group bond. Finally, the three peaks from 840 to 701 cm^−1^ were characteristic peaks of the aromatic ring. No difference can be appreciated in the spectra of the native or GO-doped membranes since the characteristic peaks of the GO are present also in the bitumen.

### 3.3. Water Contact Angle

The native bituminous membranes show a good degree of hydrophobicity by water contact angle analysis (Table 4), and this result is consistent with those reported in the literature for others for 70/100 dmm road bitumen [49]. When GO is added, there is no substantial difference in terms of wettability as a function of the GO content. This result is due to the chemical nature of GO, as it is similar to the asphaltenes of bitumen [35], and to the low percentage of GO content in the produced bituminous matrix.

### 3.4. Accelerated Weathering Studies

**Figure 3:**
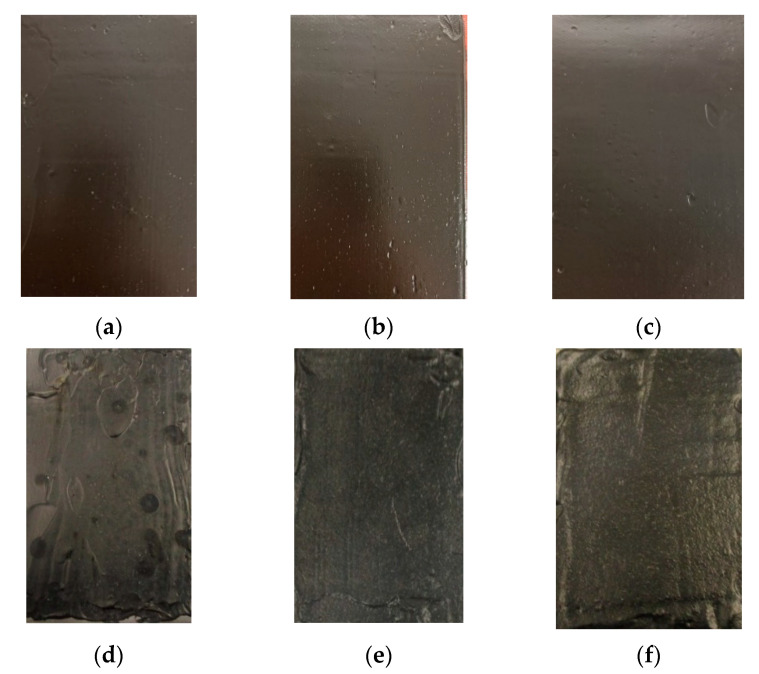
Optical images acquired just after preparation for the (**a**) native membrane, (**b**) MGLQ, and (**c**) MGHQ and after aging treatment for the (**d**) native membrane, (**e**) MGLQ, and (**f**) MGHQ.

All the freshly prepared membranes showed crack-free and regular surfaces (Figure 3a–c), while after the accelerated aging process, which was used to simulate the damages caused by exposure to atmospheric agents in medium–long periods, the native membrane showed several cracks on its surface (Figure 3d) that are not visible to the naked eyes in the GO-doped membranes (Figure 3e,f).

**Figure 4:**
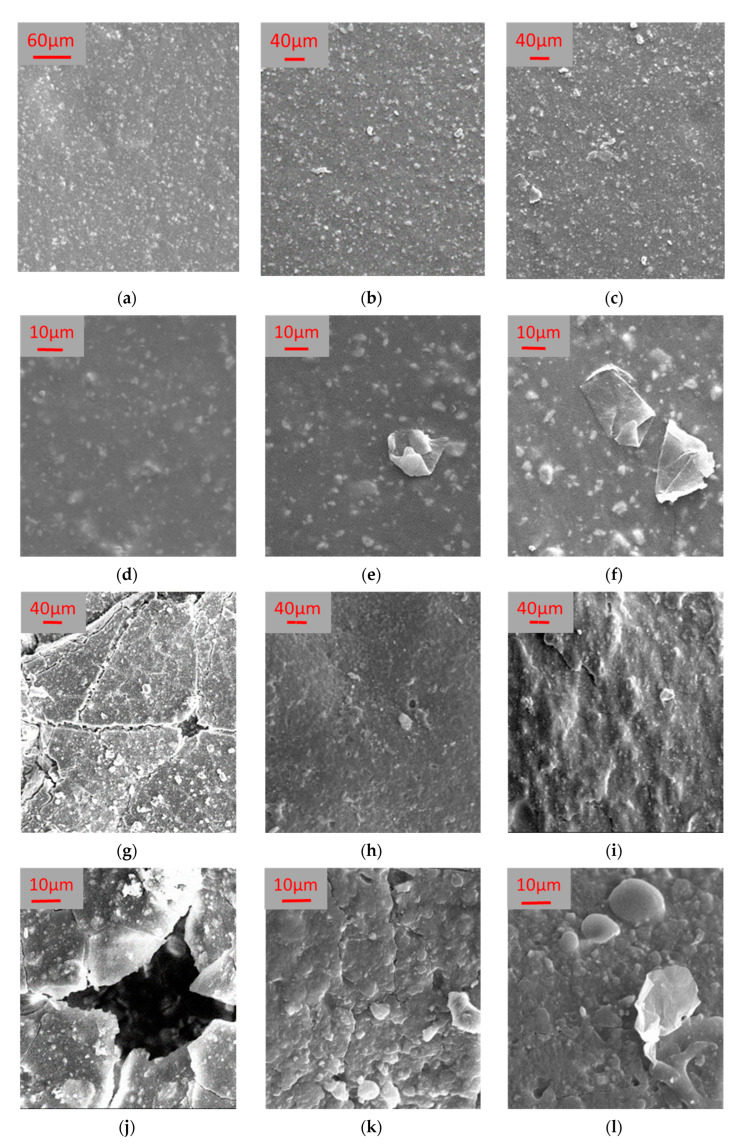
SEM images acquired just after membrane preparation for the (**a**–**c**) native membrane, the MGLQ, and MGHQ at 500× and (**d**–**f**) at 2.5k×, respectively; and after ageing treatment for the native membrane, the MGLQ, and MGHQ (**g**–**i**) at 500× and (**j**–**l**) at 2.5k×, respectively.

Figure 4 shows the SEM images acquired on the three samples, namely native, MGLQ, and MGHQ. The SEM images were acquired to evaluate membrane morphology and damage caused by accelerated aging (QUV). A dense structure was observed for all three membranes. The MGLQ and MGHQ membranes showed micrometer-sized GO platelets, but they were not found in native membrane images.

The surfaces of the freshly prepared membranes are crack-free also when analyzed by SEM at relatively high magnifications (Figure 4). Remarkably, some flakes are present on the surface and in the cross-sections of the GO-doped membranes (Figure 4b,c,e,f), and this is a qualitative proof of the incorporation of the GO in the doped membranes; meanwhile, these flakes are missing in the native membrane (Figure 4a,d).

SEM images show that cracks are also formed in the GO-doped membranes (Figure 4h,i,k,l); however, they confirm that GO-doped membranes are less prone to form cracks on their surface when exposed to accelerated aging with respect to the native membrane (Figure 4g,j). Moreover, an effect of the GO content can be qualitatively assessed, since the native membrane is very damaged, while few cracks are present on the surface of the MGLQ (Figure 4h,k), and even less are on the surface of the MGHQ (Figure 4i,l). Thus, we can state that the presence of GO in bitumen increases the oxidative thermo-stability of the bituminous membranes and also delays the degenerative phenomena generated by UV radiation [50,51,52].

### 3.5. Mechanical Performance

**Figure 5:**
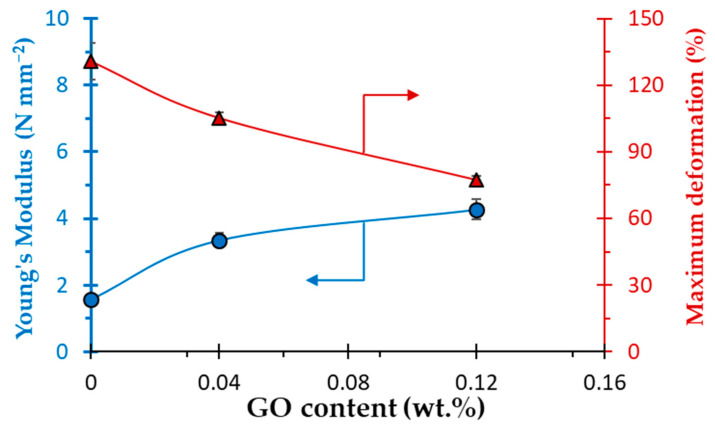
Young’s Modulus (blue circle) and maximum deformation (red triangle) as a function of the GO content in the bituminous membranes. Error bars’ dimension is smaller than or close to the dimension of the symbols.

The addition of GO leads to an increase of the Young’s Modulus and to a decrease of the maximum deformation (Figure 5). Both effects are strongest with the highest GO content, indicating that the presence of GO leads to a stiffening of the bituminous membranes as a function of the GO content. This effect is due to the establishment of interatomic interactions/forces between the matrix and the GO, as seen for other fillers [53,54,55].

### 3.6. Water Vapor Transport 

**Figure 6:**
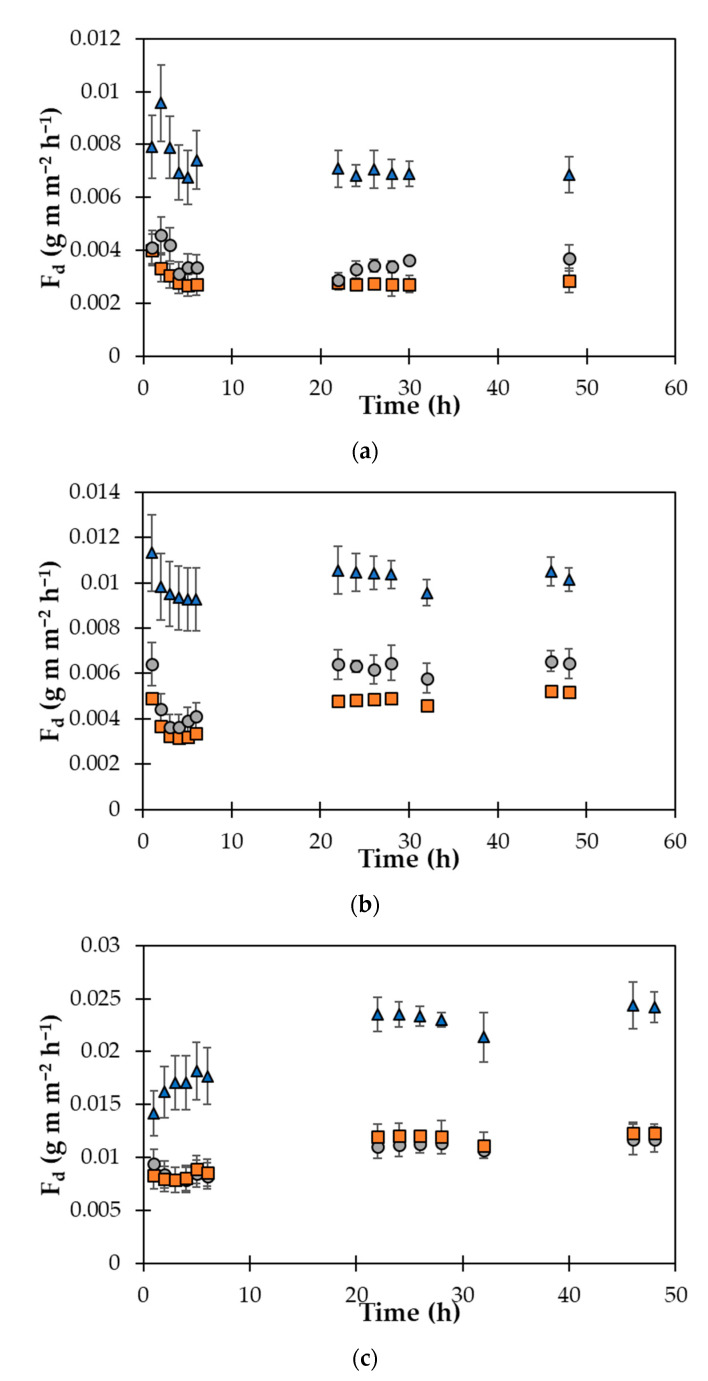
*F_d_* as function of the time for the native (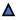), MGLQ (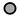), and MGHQ (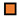) membranes at (**a**) 20, (**b**) 40, and (**c**) 60 °C.

In all the membranes proposed in this work, the water vapor transport [48] became stable after approx. 20 h, independently of the temperature of the experiment and the GO concentration (Figure 6). This is also visible in the errors associated with the measurements, which are about 15–20% in the transient region and move down to 5–10% in the stable region.

The GO concentration has a strong influence on the transmission rate; in fact, the flux is much lower in the doped membranes with respect to the neat membrane that is a model of the traditional waterproofing membrane (Table 5). This is most probably due to a drop of the water vapor diffusion coefficient caused by the presence of GO that increases the tortuosity in the membrane matrix and, above all, by increasing the activation energy of diffusion via the stiffening of the matrix (Figure 6). However, the alignment of GO platelets with respect to the flux might influence the performance. 

**Figure 7:**
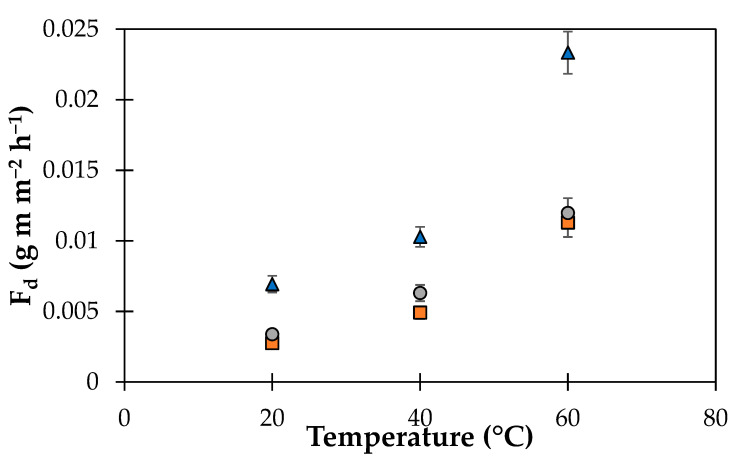
*F_d_* as a function of the temperature for the native (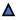), MGLQ (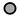), and MGHQ (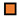) membranes.

In fact, as reported in a previous work [41], the perpendicular orientation of the GO platelets with respect to the passage of gaseous molecule increases the path of the diffusion with a consequent reduction of the permeate flow. Moreover, it has been observed that the size of GO platelets influences the length of the diffusion path from 25 to 1300 times for sizes from 100 nm to 5 microns, respectively [41]. The size and surface orientation of the GO platelets that were observed via SEM may provide a comprehensive explanation of the observed vapor transport behavior. The mass transport increases as a function of the increasing temperature in all the membranes (Figure 7), and this confirms that an activated process predominantly governs the transport. Moreover, at higher temperatures, the doped membranes present a much lower water permeation value with respect to the neat membrane.

## 4. Conclusions

The bituminous emulsions doped with graphene oxide are able to form a stable membrane with morphological and structural characteristics similar to those of the neat solution. Remarkably, the addition of the graphene oxide leads to an increase of the mechanical resistance and has anti-aging properties. These two effects become stronger with an increasing graphene oxide content; in fact, the SEM images show that the sample doped with the highest amount of GO was barely affected by the exposure to UV rays. The GO presence also affects the water permeability, which decreases in the presence of GO, but there are virtually no differences in regard to the function of the GO content. This means that both of the two formulations can be used as a waterproofing agent depending on the exposition to atmospheric agents. Moreover, viscosity measurements show that the inclusion of GO does not change the rheology of the solution; thus, no changes in the product application are foreseen. In conclusion, the introduction of GO allowed us to produce membranes that are mechanically resistant; stable to drastic conditions of oxidation, such as heat and UV rays; highly waterproof; and with a very prolonged useful life. This has a significant environmental and economic impact, since these properties can be associated with a reduced number of interventions to restore the waterproofing material and, therefore, reduce the use of raw materials and the production of special waste.

## Figures and Tables

**Table 1:** Properties of bitumen 70/100 dmm.

Bitumen Binder	Penetration at 25 °C ^1^ (dmm)	Softening Point ^2^ (°C)	Viscosity at 135 °C ^3^ (mm^2^ s^−1^)
Alma Petroli S.p.A.	70–100	46–54	min 230

^1, 2, 3^ These properties were evaluated according to the following standard methods: ^1^ EN 1426, ^2^ EN 1427, and ^3^ EN 12595.

**Table 2:** Properties of the native bitumen emulsion.

Parameter	Test Method	Thresholds ^1^	This Work Value
Water content	EN 1428	45 ± 2%	43.0%
Binder content	EN 1431	55 ± 2%	57.0%
Homogeneity	EN 1429	max 0.2%	0.05%
Sedimentation at 7 days	EN 12847	max 10%	3%
Cement mix	EN 12848	<2 g	<2 g

^1^ Range of typical value for bitumen emulsion.

**Table 3:** Viscosity measurement of the bitumen emulsion samples.

Sample	Test Method	Value at 20 °C (Poise)
Native	ASTM D 2196	680 ± 20
MGLQ	ASTM D 2196	680 ± 20
MGHQ	ASTM D 2196	700 ± 20

**Table 4:** Physical properties of the membranes.

Sample	GO Content (wt.%)	WCA (°)	Thickness (mm)
Native	0	105.6 ± 3.6	1.00 ± 0.05
MGLQ	0.04	108.1 ± 4.2	0.91 ± 0.01
MGHQ	0.12	106.4 ± 3.1	0.92 ± 0.03

**Table 5:** Mass fluxes of water vapor through the self-standing bituminous membranes multiplied by membrane thickness, *F_d_*, measured at 20, 40, and 60 °C and as an average of the experimental data taken in the range of 20–48 h.

	*F_d_* (g m m^−2^ h^−1^)		
Membrane	20 (°C)	40 (°C)	60 (°C)
Native	0.007 ± 0.001	0.010 ± 0.001	0.023 ± 0.001
MGLQ	0.003 ± 0.001	0.005 ± 0.001	0.011 ± 0.001
MGHQ	0.003 ± 0.001	0.006 ± 0.001	0.012 ± 0.001

## Data Availability

Not applicable.
